# Perceptions about screening for prostate cancer using genetic lifetime risk assessment: a qualitative study

**DOI:** 10.1186/s12875-018-0717-6

**Published:** 2018-02-17

**Authors:** Pia Kirkegaard, Adrian Edwards, Trine Laura Overgaard Nielsen, Torben Falck Ørntoft, Karina Dalsgaard Sørensen, Michael Borre, Flemming Bro

**Affiliations:** 1 Department of Public Health Programmes, Randers Regional Hospital, Skovlyvej 15, 8930 Randers NO, Denmark; 2 Division of Population Medicine, Cardiff University, Cardiff, UK; 3 Research Unit for General Practice, Department of Public Health, Aarhus University, Aarhus C, Denmark; 4 Department of Molecular Medicine, Aarhus University Hospital, Aarhus N, Denmark; 5 Department of Clinical Medicine, Aarhus University Hospital, Aarhus N, Denmark

**Keywords:** Cancer, Genetics, Cancer, Screening and prevention, Decision making, Primary health care, Risk, Behaviors

## Abstract

**Background:**

Most health authorities do not recommend screening for prostate cancer with PSA tests in asymptomatic patients who are not at increased risk. However, opportunistic screening for prostate cancer is still wanted by many patients and it is widely used in primary care clinics, with potential for overdiagnosis and overtreatment. Better tools for risk assessment have been called for, to better target such opportunistic screening. Our aim was to explore perceptions about prostate cancer risk and subsequent opportunistic screening among patients who were not at increased risk of prostate cancer after a first PSA test plus a genetic lifetime risk assessment.

**Methods:**

We undertook semi-structured patient interviews with recording and verbatim transcription of interviews. Data were analysed thematically.

**Results:**

Three themes were identified: uncertainty of the nature of prostate cancer; perceived benefits of testing; and conflicting public health recommendations. Prostate cancer was spoken of as an inescapable risk in older age. The aphorism “you die with it, not from it” was prominent in the interviews but patients focused on the benefits of testing now rather than the future risks associated with treatment relating to potential overdiagnosis. Many expressed frustration with perceived mixed messages about early detection of cancer, in which on one side men feel that they are encouraged to seek medical testing to act responsibly regarding the most common cancer disease in men, and on the other side they are asked to refrain from opportunistic testing for prostate cancer. Taken together, personal risks of prostate cancer were perceived as high in spite of a normal PSA test and a genetic lifetime risk assessment showing no increased risk.

**Conclusion:**

Patients saw prostate cancer risk as high and increasing with age. They focused on the perceived benefit of early detection using PSA testing. It was also commonly acknowledged that most cases are indolent causing no symptoms and not shortening life expectancy. There was a frustration with mixed messages about the benefit of early detection and risk of overdiagnosis. These men’s genetic lifetime risk assessment showing no increased risk did not appear to influence current intentions to get PSA testing in the future.

**Electronic supplementary material:**

The online version of this article (10.1186/s12875-018-0717-6) contains supplementary material, which is available to authorized users.

## Background

Cancer has been established in public health discourses as one of the most dreaded diseases in modern society [1]. Strong cultural metaphors include ‘rot that eats you up’ and ‘war between good and bad cells in the body’ [2, 3]. Cancer patient associations in Denmark are well supported by the public, are highly visible in the media, and early detection of cancer has caught the attention of both politicians and citizens [4, 5].

Prostate cancer (PCa) is the most commonly diagnosed cancer in men and the second leading cause of cancer deaths in men in the Western world [6]. Known risk factors are old age, family disposition, and black ethnicity [7]. Approximately 40–50% of all 50-year-old men and more than 80% of 80-year-old men carry an undiagnosed PCa which does not cause premature death [8]. Although PCa is one of the most heritable of all cancers, the aetiology of the disease is still poorly understood [9, 10]. The benefit of treatment is limited as PCa is indolent and non-aggressive in up to 90% of cases and treatment is often associated with side-effects such as incontinence and erectile dysfunction [11]. The Prostate Specific Antigen (PSA) test is still the best biomarker for detection of PCa but the test has poor specificity [12, 13]. A high level of PSA may indicate PCa but PSA levels also increase with age due to benign prostatic hyperplasia [14]. Opportunistic PSA testing – defined as medical testing or screening that makes use of an opportunity engendered by other presentations and/or tests to which a patient is accustomed or has already given consent – is common [15]. The risk of overdiagnosis, defined as disease that ultimately will not cause individuals to experience symptoms or early death, has been estimated at 60% or more of all cases of PCa [16]. The benefit of early detection is considered to outweigh the risk of overtreatment only in patients with a family history of PCa [17, 18]. In the context of poor general knowledge among lay people about the pitfalls of PSA testing, the PSA test is still widely requested by patients [19–21].

Several initiatives have been made to raise awareness about the benefits and harms of PSA testing in patients who are not at increased risk of PCa, including the development of decision aids to support the dialogue between doctor and patient [22–24]. Decision aids have demonstrated effects on patient knowledge and satisfaction with decisions, but effects on intention to request PSA testing or actual PSA testing behaviour are more equivocal [25, 26]. Interventions using genetic lifetime risk assessment for PCa as a complementary tool to support active decisions about PSA testing have been developed and the technology is evolving rapidly [27–30]. However, genetic lifetime risk assessment of PCa still awaits implementation in general practice [31].

The aim of this study was to explore perceptions about PCa risk and subsequent opportunistic screening among patients who were not at increased risk of PCa after a first normal PSA test with an average genetic lifetime risk assessment.

## Methods

### Study design

This was a qualitative study with patients from general practice in Denmark. It was embedded in an international translational study (the Molpros Study) about molecular prediction of PCa risk and aggressiveness, in which a clinical tool for genetic lifetime risk assessment of PCa was developed and tested in general practice (the ProCaRis Study) [32, 33].

### Participants and recruitment

In a three-month pilot trial period, five local general practices were given access to a clinical tool for genetic lifetime risk assessment of being diagnosed with PCa. The general practices offered a genetic lifetime risk assessment to any patients requesting a PSA test if they had no previous or current PCa diagnosis and no previously measured abnormal PSA-values (> 4.0 mmol/L). Depending on the result of the PSA test and the risk assessment, three types of reply were provided from the research laboratory in the Molpros Study to the general practices: 1) “According to a genetic lifetime risk assessment, this patient has a high genetic lifetime risk of being diagnosed with prostate cancer. The patient may benefit from regular individual screening with PSA tests”, 2) “According to a genetic lifetime risk assessment, this patient has an average lifetime risk of being diagnosed with prostate cancer. The patient is unlikely to benefit from regular PSA tests unless he gets symptoms associated with prostate cancer”, and 3) “The genetic risk assessment could not determine the genetic lifetime risk of this patient. The patient may benefit from regular individual screening with PSA tests”. The GPs conveyed the result to patients according to usual practice for giving test results.

We focused here on the second group as this would be expected to be the largest group in which changes in PSA testing might lead to more ‘appropriate’ (and less) testing if genetic risk assessment were to affect such subsequent behaviours [33]. According to laboratory data, twelve patients had a normal PSA test result and an average genetic lifetime risk of being diagnosed with PCa. The general practices obtained consent from these patients to disclose their telephone number to the researcher (PK) who contacted them within 4 weeks to invite them to participate in an interview study. All invited patients received written material about the study before the interview.

### Data analysis

We developed a semi-structured interview guide for the interviews, which took place at the patients’ homes [34]. The guide included the following topics: reason for visiting the doctor when the first PSA test had been taken, symptoms, knowledge about PCa, PSA testing and genetic risk assessment, attitude to PSA screening, intentions to get future PSA tests, risk perception, and experiences with the healthcare system. An additional file shows the semi-structured interview guide [Additional file 1, Interview guide]. Each interview was conducted by PK or TLON who are experienced research interviewers. Each interview lasted between 45 and 60 min. The interviews were recorded and transcribed verbatim by PK and TLON. Analysis was undertaken in an inductive thematic manner in which all datasets were read and re-read in search for emerging themes (‘funnelling’) [35], using Nvivo 9 software, and emerging themes were discussed regularly among PK, FB and AE. The codes were arranged into patterns which were condensed into themes and discussed among all authors in order to challenge the analytical insights, and this iterative process continued until consensus was reached and a coherent analysis was developed.

### Research ethics

The study followed the Statements on Ethics of the American Anthropological Association [36] and was approved by the Danish National Committee on Health Research Ethics (journal no. 1–10–72-43-12). The study was approved by the Danish Data Protection Agency (journal no. 2011–41–6904) and collection of data handled according to their guidelines. The names that appear in the paper are pseudonyms.

## Results

All 12 patients with normal PSA and average lifetime risk of being diagnosed with PCa accepted to participate in the interview study (Additional file 2, Table S1). In the analysis, we identified and explored three themes in the accounts: uncertainty of the ‘true nature’ of PCa, the perceived benefits of testing, and frustration about public health recommendations.

### “It’s Tricky. Some Get It and Live With It”: Uncertainty of the ‘true’ nature of prostate cancer

A common aphorism about PCa is that “you die with it, not from it”. This implies that the risk of having (an indolent) PCa is high, but the risk of dying from (an aggressive) PCa is low. The patients in our study also referred to PCa as “tricky” because the level of aggressiveness tends to vary from one case to another. This was explained by Johan, aged 61: “It’s tricky. Some get it and live with it. They have a harmless version of it, and they don’t even know it. Others get a severe diagnosis, and then they just die. They wither away. It goes into the bones and makes them crack.” His father was diagnosed with PCa after a fall that broke his back. The course of his disease was short and “ugly” as Johan put it. His father subsequently died of PCa.

Another patient, Jens, aged 55, also referred to the unpredictable nature of PCa and mentioned a popular Danish politician, Svend Auken, who was diagnosed with PCa in 2004: “You know, you die with it, not from it. But there are cases where you die quickly. Svend Auken, for instance. That’s brutal.” Other patients also mentioned the Danish TV and cinema documentary ‘Svend’, which was released in 2011. The film was originally intended to portray Svend Auken’s political endeavours, but it also became a portrait of his fight with PCa that eventually ended his life at the age of 66 in 2009.

Some patients particularly stressed time as a key factor in defining PCa as a deadly disease or as a chronic condition. Old age is a time of competing health risks, as expressed in a humorous tone of voice by Johannes, aged 62: “I don’t give it much thought. You get it or you don’t. You get really sick or you live with it and die of something else. Old age is a killer!”.

The interviewed patients referred to PCa as an expected disease in older age. The youngest patient, Peter, 34 years old, requested the PSA test because his father got PCa in his fifties and died from it a few years ago. “If PCa strikes when the patient is young, the cancer is more severe”, Peter said. He said about the PSA test and his doctor: “I guess I get the test to be on the safe side, especially if the doctor thinks that it is a good idea. And I was really worried, so he thought it was a good idea.” Peter expected to worry even more as he grew older, particularly when reaching the age at which his father had died.

### “I’m Not the Type to Say ‘No Thanks’ ”: The perceived benefits of testing

Most interviewed patients reported that they perceived early diagnosis before getting symptoms as better than diagnosis “in due course” as one patient put it. During the interview, the patients were asked to choose between a hypothetical early diagnosis of a non-severe PCa that would not cause early death, or ‘blissful ignorance’ about it; most patients expressed that they would want to know. One patient, Regnar, aged 58, exclaimed: “How do you mean? Of course everybody wants to know! I would want to know about it! Then they could keep an eye on me. Even though I couldn’t do anything about it, I’d still like to know what is going on.” Gunnar, aged 67, said: “It is always better to know. If you know, you can do something. If you don’t know, then nothing. My doctor says’ you don’t die from it, you live with it’. But still, I want the PSA test so something can be done before it’s too late”.

Frede, aged 60, questioned the imperative of early diagnosis. “You don’t have to know all about all the faults and flaws. It will just make you sick if you go around and worry about things that might not happen anyway. Everybody has cancer in some form, they say. Body cells mutate and get flawed. Feel good and be in good shape, that’s the most important thing. Stay mentally healthy. But if my doctor advises me to get the PSA test, I’m not the type to say’ no thanks’. He knows what he is doing and he knows me”.

No patients showed any particular interest or knowledge about genetic lifetime risk assessment (despite all having had one). When asked about the consequences for him of a ‘normal/average genetic lifetime risk’ and his intention to get a PSA test, Peter, aged 34, said: “They know so much, and they come up with something new all the time. I don’t know how much I can trust it, but the more you get tested, the more the doctor knows about your health, right?” Johannes, aged 62, put it this way: “Things change. Well, I might as well get the PSA test again if I’m in the consultation anyway. It feels good to go with the flow. It’s just a risk assessment, right, not an insurance policy. You do what you can do. And this is what you can do”.

### “It’s Not Easy to Satisfy Everyone”: public health recommendations

Frustration about conflicting public health recommendations and perceived mixed messages was frequently expressed in the interviews. Harald, aged 55, reasoned: “We men are told again and again to see a doctor more often because men live harder and die earlier. It’s not easy to satisfy everyone. When you go to the doctor, he argues that the PSA test is used too frequently, and there are side effects. It’s not rational, is it?”.

John, aged 61, also pointed out a “reprimanding” tone in public health recommendations to men about taking care of their health, which was contested when they finally consulted their doctor: “Sometimes we are accused of not taking care of ourselves. But listen, I have never had a day off sick. My health is excellent. I go to check-ups due to high cholesterol and erysipelas, a skin infection. But I couldn’t get a PSA test because the doctor said it was unnecessary. Now my father, he is 86, and got prostate cancer. I think she [the doctor] should have listened to me”.

Regnar, aged 58, complained about his doctor: “She is not easy to convince about the prostate test [PSA test]. She says that it will become too high eventually, when I get old enough, so if I get the test repeatedly, they will just find a cancer sooner or later. I can’t see the logic in what she’s saying. Finding it early is better. So I request it once a year. And I get it”.

Perceptions about cancer and early diagnosis were sometimes explained as a result of prominent public cancer awareness campaigns and personal or celebrity stories about cancer in the media. Jørgen, aged 59, recalled the international “Movember” campaign and a national health campaign by the Danish Cancer Society about men’s health care-seeking behavior: “The one with the moustache and the one with ‘Real men visit their doctor’. You better go! You see health recommendations targeting men everywhere. We drink and smoke too much, drive too fast on the highway, exercise too little. We should have our health checked”.

The cancer story of Svend Auken mentioned above was also highlighted as a driver of public interest about cancer. Frede, aged 60, said: “It’s a Svend Auken effect, if you may call it so. You see it every time a famous person gets a severe disease – now everybody’s afraid of prostate cancer.”

Some patients discussed possible male discrimination in cancer research and screening. Jens, aged 55, said (in a humorous tone of voice): “My wife is right when she points out that women are invited for screening, but men are not. Isn’t it about time they invite us?” John, aged 61, also pointed out that women get screened, but men do not: “So when you hear that women are offered screening for cancer, you start wondering why men are denied screening for cancer ─ especially when you know for a fact that cancer is so common”.

## Discussion

Patients in our study spoke of PCa as a “natural” part of getting older, and the aphorism “you die with it, not from it” was often mentioned in the interviews. These patients spoke of the PSA test as a crucial prerequisite for cancer detection and they focused on the perceived benefits of testing now rather than the risks associated with treatment in the future. Frustration about conflicting public health recommendations was frequently expressed because public health recommendations for men to take care of their health (by undergoing various tests) were contested in the clinic when the patients finally consulted their doctor, some of whom reportedly advised them not to take a PSA test. During the interviews, the patients did not take into account the normal/average result of their genetic lifetime risk assessment when reasoning about future PSA testing for themselves or for other men.

### Limitations

The general practitioners and practice nurses were carefully instructed to inform the patients about the risk and benefits about PSA testing and genetic risk assessment but how information was given and results communicated remain unknown. The patients were not concerned about the risks of overtreatment and focused instead on the perceived benefits of early detection of PCa. This is surprising given the purpose of a genetic lifetime risk assessment which is to reduce risk of overtreatment and side-effects associated with treatment. It suggests that information in the consultation about the benefits and risks of PSA testing, and the benefits and risks of genetic lifetime risk assessment were mixed together, and it underscores the complexities in general practice regarding communication about risk of future disease. Second, there was a time restriction on the recruitment phase because the testing of the clinical tool in the five general practices followed a protocol involving a wide range of stakeholders in the translational study, inside which the interview study was nested [32]. It limited the number of eligible patients for the interview study, constraining opportunity to test or reach saturation or ‘information power’ [37]. We responded to this by refining the research question during the analytical process, based on the empirical data, so that conclusions about individuals (male patients) from a specified group (requesting PSA testing) sharing similar experiences not previously described (normal PSA value in combination with a genetic lifetime risk assessment showing average risk of being diagnosed with PCa) could be drawn [35].

Third, the overall methodological approach to testing the clinical tool in the ProCaRis Study was to mimic the way in which clinical tools are implemented in everyday clinical practice where the success of a new non-diagnostic clinical tools also depends on convenience and ability to ‘fit’ with existing procedures. Hence the general practices were not asked to make any further records about invited patients who declined to receive a genetic lifetime risk assessment or potential participants who were not invited due to time pressure, omission, etc. Thus we were not able to assess potential selection bias in the general practices’ recruitment of patients. However, the strength of our study was that it reflects how new tests and laboratory analyses are introduced in general practices because we only “minimally disrupted” the general practices in their interaction with patients.

### Comparison with existing literature

We found that PCa was framed as a ‘natural’ part of becoming old, a ‘risk of age’ and blurring the lines between ageing as a natural process of ‘biomorphism’, and pathology [38]. Estimates of individual PCa risk and diagnostic means to detect a PCa were welcomed even though the uncertainty of PCa was not replaced by certainty when using the PSA test or the genetic risk assessment. This is consistent with research showing that the diagnostic uncertainty of the PSA test may be acknowledged among patients as a ‘maybe test’ and the decision to undertake PSA testing can be fraught with uncertainty both before and after testing, even if the test result turns out to be normal [39–41]. In our study, the diagnostic uncertainty was also acknowledged as a major pitfall, but the perceived opportunity to get a ‘look inside’ was often felt to outweigh the risks of getting a diagnosis of an indolent PCa.

According to the patients, the decision to undergo PSA testing was patient-centered or even patient-led in most cases. They felt they had a right to be screened for cancer implying that the lack of systematic screening for more cancer types among men is a poor political decision. This suggests that the political aspect outweighed apparent health risks. It is supported by studies showing that sometimes the desire for a PSA test is unaffected by information from the GP about the pitfalls of PSA testing while in other cases, recommendations from the GP, family, or friends have a powerful influence on decisions about PSA testing [20, 42].

Regarding lifetime risk assessment it has been suggested that a seeming lack of interest in information about possible implications of (genetic) risk assessment could be seen against a backdrop of trust in health care professionals [43, 44]. Many patients decide to let their GP make the decision for them about testing in preference-sensitive cases. This may be both rational and beneficial but it can be difficult for the GP to discriminate between patients who make an informed decision about letting the GP decide, and patients who do not realise that they have a choice to make [45]. This challenges the aspiration for informed decision-making about new health technology and testing opportunities.

Studies about attitudes to genetic risk assessment of PCa have found low levels of worry and a high interest in genetic testing [46–48]. In our study, the genetic lifetime risk assessment did not receive any attention, and the patients did not seem to discriminate between the prognostic value of the PSA test here and now, and the lifetime perspective of a risk assessment. This accords with research showing that blood testing is perceived as relatively common and harmless by most patients and the use of blood testing as part of a diagnostic procedure has become routine in so many clinical encounters that it is now ‘mundane’ and unquestioned, and that this over-rides the details of which test may be involved [49]. It is a common perception among most patients that PSA testing and treatment of PCa are relatively ‘straightforward’, similar to measurement of cholesterol and treatment with risk-reducing medications. A PSA test might not be just a simple blood test and patients may not anticipate the ‘commitment’ to treatment in case of a positive result [41].

As in other contemporary Western societies, the dominant health discourse in Denmark is characterised by a moral imperative to optimise health and reduce exposure to health risks [50]. The patients in our study struggled to balance the moral imperative to seek health care in time and yet also avoid unnecessary tests with the risk of overdiagnosis. Our findings substantiate the gap between the current public health discourses about proactive healthcare-seeking, and the clinical pitfalls of early detection of PCa [51]. This gap might hinder attempts to reduce PSA testing in patients who are not considered to be at high risk of PCa. Informed decision-making in the consultation is crucial to ensure that patients understand the implications of opportunistic testing, but more research is needed to fully understand the gap, and how best to support discussion and decision-making.

## Conclusion

PCa was perceived as a “natural” part of getting older and the patients saw opportunistic PSA testing as a means to reduce the risk of PCa. They were aware of the risk of overdiagnosis but focused on the perceived benefits of testing. The clinical recommendation of no opportunistic screening conflicted with a societal health imperative to seek early detection of cancer. The result of the genetic lifetime risk assessment was considered ‘just another test’ result which did not influence their current intentions to get PSA testing in the future. The results are important to take into consideration before implementing new tools for genetic lifetime risk assessments in general practice.

## Additional files

Additional file 1: Interview guide used in the study. (PDF 60 kb)

Additional file 2: Table S1. Participant characteristics including age, marital status, education, and number of relatives with PCa. (PDF 325 kb)

## Abbreviations

GP: General practitioner
PCa: Prostate cancer
PSA: Prostate-specific antigen
