# Supplementary material for: Perceptions about screening for prostate cancer using genetic lifetime risk assessment: a qualitative study
Source: BMC Fam Pract. 2018 Feb 17;19:32. doi: 10.1186/s12875-018-0717-6 (PMC5816534; doi:10.1186/s12875-018-0717-6)
Supplement: Supplementary file 1 — Interview guide used in the study. (PDF 60 kb) [file 12875_2018_717_MOESM1_ESM.pdf]

Interview guide

Opening
Everyday life: work/daily activities, family/social life, leisure.
Information about the course of the interview, audio-taping, anonymity.

1. Knowledge and sources of knowledge

1a. From where do you mostly get information about health and disease?

1b. Could you tell me what is prostate cancer?

1c. Could you tell me what is a PSA test?

1d. What are the treatment options if a person gets prostate cancer?

1e. Do you know / have you heard about anyone with prostate cancer?

1f. Do you know / have you heard about anyone who has had a PSA test?

2. Reasons for visiting the doctor

2a. How often do you go to visit your doctor, and on what occasions?

2b. Why did you get a PSA test last time you went to see your doctor?

2c. Had you ever had a PSA test before?

2d. Could you tell me what happened during the consultation – what did you say to your doctor, and what did he/she say?

2e. Did you talk about your individual risk of getting a prostate cancer diagnosis now or in the future?

2f. Did you get any other tests than a PSA test?

3. Results from the consultation

3a. What was your PSA level?

3b. Did you get any other lab results?

3c. What do you think about your risk of getting prostate cancer in the future?

3d. Do you wish to get a PSA test again?

3e. Have you talked to your spouse/family/friends about the lab result(s)?

4. Attitude to cancer screening

4a. Would you recommend your (brother/son/friend) to get screened regularly for prostate cancer with a PSA test?

4b. Should everybody get screened for cancer? Who should (not) and why?

4c. What do you think about the current use of resources and attention allocated to early detection of cancer?

4d. Do you think the healthcare system should spend more money or less money on early detection of cancer (prostate cancer), compared with early detection of other common diseases?

Round-off
Recapitulation, additional comments.
