# Supplementary material for: Perceptions about screening for prostate cancer using genetic lifetime risk assessment: a qualitative study
Source: BMC Fam Pract. 2018 Feb 17;19:32. doi: 10.1186/s12875-018-0717-6 (PMC5816534; doi:10.1186/s12875-018-0717-6)
Supplement: Supplementary file 2 — Participant characteristics including age, marital status, education, and number of relatives with PCa. (PDF 325 kb) [file 12875_2018_717_MOESM2_ESM.pdf]

Table S1. Participant characteristics including age, marital status, education, and number of relatives with PCa

Participant	Age	Marital status	Educational level	Relatives with PCa
Jørgen	59	Married	Post-secondary non-tertiary	1
Jens	55	Married	Short-cycle tertiary	1
John	61	Single	Master or equivalent	1
Peter	34	Single	Post-secondary non-tertiary	1
Harald	55	Married	Master or equivalent	0
Bent	51	Co-habiting	Master or equivalent	0
Johan	61	Single	Short-cycle tertiary	1
Hans	58	Married	Post-secondary non-tertiary	0
Regnar	58	Married	Upper secondary	0
Gunnar	67	Married	Post-secondary non-tertiary	1
Frede	60	Single	Post-secondary non-tertiary	1
Johannes	62	Married	Post-secondary non-tertiary	0
